# Transcriptional Upregulation of HERV-*env* Genes Under Simulated Microgravity

**DOI:** 10.3390/v17030306

**Published:** 2025-02-23

**Authors:** Seyedesomaye Jasemi, Elena Rita Simula, Antonella Pantaleo, Leonardo A. Sechi

**Affiliations:** 1 Department of Biomedical Sciences, Division of Microbiology and Virology, University of Sassari, 07100 Sassari, Italy; sjasemi@uniss.it (S.J.); simulaelena@gmail.com (E.R.S.); apantaleo@uniss.it (A.P.); 2 Struttura Complessa Microbiologia e Virologia, Azienda Ospedaliera Universitaria Sassari, 07100 Sassari, Italy

**Keywords:** HERV gene expression, cytokine expression genes, microgravity simulation

## Abstract

Human endogenous retroviruses (HERVs) constitute about 8% of the human genome. The overexpression of HERVs has been detected in various inflammatory disorders like neuro-inflammation disorders and cancer. Interestingly, it has been reported that stress conditions facilitate HERV expression. Space travel exposes astronauts to microgravity environments (a stress condition), which may result in the activation of HERVs and might influence pathogenic outcomes during and after space flight. This study aimed to elucidate the transcriptional activity of three HERV families (W, K, and H) and cytokine genes (*IL-1*, *IL-6*, and *TNF*-α) in different cell lines under microgravity (μg) conditions and compare them with the results obtained under normal gravity (ng; 1g). We evaluated the expression of HERVs (HERV-K *env*, HERV-K *gag*, HERV-W *env*, and HERV-H *env*) and cytokine gene expression (*IL-1*, *IL-6*, and *TNF*-α) in neuroblastoma (SH-SY5Y), HEp-2, and Caco-2 cell lines under simulated μg and 1g conditions. In SH-SY5Y cells, the expression level of the *IL-1*, *IL-6*, HERV-H *env*, HERV-K *env*, HERV-K *gag*, and HERV-W *env* genes was significantly increased when exposed to short-term μg (3 and 6 h). The expression of *TNF*-α remained unchanged throughout all time points. Additionally, in Caco-2 cells, the expression of the HERV-K *env*, HERV-K *gag*, and *IL-1* genes was significantly higher after 6 h of incubation in μg conditions compared to 1g. There was no statistically significant difference in the expression levels of the HERV-W *env*, HERV-H *env*, *IL6*, and *TNF*-α genes between the μg and 1g conditions. Moreover, in HEp-2 cells, the expression of the *IL-1*, *IL6*, *TNF*-α, HERV-H *env*, HERV-K *env*, HERV-K *gag*, and HERV-W *env* genes significantly increased following short-term incubation in μg (3 h, 6 h) and then decreased to levels comparable to those observed in the 1g condition. Taken together, the dysregulation of cytokine and HERV gene expression was observed under the simulated μg condition. The patterns of these dysregulations varied throughout cell lines, which demands further investigation for human health protection in space.

## 1. Introduction

Human endogenous retroviruses (HERVs) make up about 8% of the human genome [1]. They integrated into the human genome during evolution [2]. Due to a variety of genomic rearrangements, including mutations and epigenetic regulatory mechanisms, these viral elements are transcriptionally silenced under normal physiological conditions [3]; however, the aberrant expression of HERVs has been associated with various pro-inflammatory diseases, including neuro-inflammatory disorders and different types of cancers [4,5,6].

Structurally, HERV sequences constitute of four genes including *gag*, *pro*, *pol*, and *env* genes flanked by two long terminal repeats (LTRs). Briefly, *env* encodes the transmembrane (TM) and Env surface (SU) subunits and is subject to stronger selection pressures because of its increased exposure to the host immune system. *Pro* and *pol* specify the enzymes protease (PR), reverse transcriptase (RT), and integrase (IN); and *gag* encodes the structural components of the matrix, capsid, and nucleocapsid [7]. 

The abnormal upregulation of some HERVs can trigger the immune system response, leading to immune system dysregulation and inflammation [8]. For instance, it has been demonstrated that HERV-K(HML-2) Env activates TLR7/8 in neurons and microglia, which is associated with neurodegeneration through a pro-inflammatory response [9]. Similarly, the interaction between HERV-W envelope protein and TLRs triggers a strong pro-inflammatory response and the release of many cytokines, including *TNF-a*, *IL-1b*, and *IL-6* [10]. On the other hand, it has been demonstrated that a portion of HERV Env proteins, like the conserved component immune-suppressive domain (ISD) of HERVH-Env, is implicated in immune-suppressive mechanisms [11]. Interestingly, evidence suggests a bidirectional link between ERV expression and inflammation, where HERVs have the ability to induce an inflammatory response and also be activated by it [12]. This feedback loop exacerbates inflammation and contributes to disease progression [13].

Studies have consistently presented evidence that the immune system is among the most affected systems during microgravity in space or simulated microgravity on Earth, characterized by impaired immune cell function, reduced lymphocyte activity, and increased inflammation [14,15]. Despite extensive studies on immune system dysregulation under microgravity, HERV expression has not been investigated and is still unclear.

Given the well-established link between immune system dysregulation and HERV gene expression [10,16], as well as the known effects of microgravity on immune functions [15,16], it is of great interest to investigate the possible effects of microgravity on HERV gene expression. Understanding these effects may reveal key mechanisms underlying the inflammatory responses observed under altered gravitational conditions. As HERV gene expression has been demonstrated in a different cell line [5,17,18,19] and is overexpressed in neurodegenerative diseases and cancer [4,5,6], we selected three specific cell lines (neuroblastoma SH-SY5Y, and cancerous Caco-2 and HEp2) for this study. Each of these cell lines represents different biological contexts and immune responses. This approach may allow us to gain comprehensive knowledge of HERV inflammatory dysregulation mediated by microgravity, with a focus on the cell lines’ roles in cancer and neuro-inflammation disorder. They include the human neuroblastoma cell line SH-SY5Y, which is widely used in neurotoxicity research, producing various pro-inflammatory cytokines such as *IL-1β*, *IL-6*, and *TNF-α*, as well as the cancer cell lines Caco-2 (colorectal adenocarcinoma) and HEp-2 (human epithelial cells derived from a larynx carcinoma). This study aimed to investigate the transcriptional activity of HERVs and pro-inflammatory cytokine genes in three human cell lines (SH-SY5Y, Caco2, and HEp2) under simulated microgravity conditions. The findings could provide useful insights into whether microgravity modifies HERV expression and lead to a better understanding of immune system dynamics in microgravity environments.

## 2. Materials and Methods

### 2.1. Cell Cultures

Three distinct cell lines were investigated: human neuroblastoma SH-SY5Y cells (CRL-2266, ATCC, Rockville, MD, USA), human laryngeal carcinoma Hep-2 cells [CCL-23, ATCC, USA], and human colorectal adenocarcinoma Caco-2 cells [HTB-37, ATCC, USA]. The cell lines were cultured in Dulbecco’s Modified Eagle’s Medium/F12 (DMEM/F12, Sigma-Aldrich, St. Louis, MI, USA), supplemented with 10% heat-inactivated fetal bovine serum (FBS) and a penicillin/streptomycin solution (100 U/mL and 100 µg/mL, respectively; both from Sigma-Aldrich, St. Louis, Missouri, USA). The cells were incubated at 37 °C in a humidified atmosphere containing 5% CO_2_ and sub-cultured every 3–4 days. For the experimental setup, 1 × 10^5^ cells of each cell line were seeded into T12.5 flasks (*n* = 18 flasks for each cell line) containing DMEM/F12 with 10% FBS, 100 U/mL penicillin, and 100 µg/mL streptomycin. The cells were incubated at 37 °C in a humidified incubator with 5% CO_2_ until 90% confluence was reached.

### 2.2. Simulated Microgravity

To verify whether cytokine and HERV gene expression could be affected by microgravity conditions, experiments were conducted using a 3D random positioning machine (RPM, Fokker Space, Amersfoort, The Netherlands) at the laboratory of the Department of Biomedical Sciences, University of Sassari, Sardinia, Italy [20]. This instrument creates rotation along three axes, simulating the microgravity conditions (μg) found in aerospace environments.

In this experiment, eight flasks were placed under the RPM device (microgravity conditions), while an equal number of flasks were kept at 37 °C in a humidified incubator with 5% CO_2_, representing the normal gravity condition (1g), as controls. Cells were collected at four time points: 3 h (*n* = 2 flasks for µg and 2 for 1g), 6 h (*n* = 2 flasks for µg and 2 for 1g), 12 h (*n* = 2 flasks for µg and 2 for 1g), and 24 h (*n* = 2 flasks for µg and 2 for 1g). At the end of each incubation period, the cells were immediately harvested using trypsin–EDTA (Sigma-Aldrich, St. Louis, MI, USA) for subsequent RNA extraction and cDNA production.

### 2.3. RNA Extraction, Reverse Transcription, and Real-Time PCR

To investigate the expression of HERV- and cytokine-related genes in different cell lines following incubation in 1g and RPM-simulated μg conditions at various time points, we conducted quantitative real-time PCR (qRT-PCR) analyses.

Total RNA was isolated using the Qiagen RNeasy Mini Kit (Qiagen, Hilden, Germany), following the manufacturer’s instructions. Subsequently, complementary DNA (cDNA) was synthesized from 2 µg of total RNA using the RevertAid RT Kit (Thermo Fisher Scientific, Waltham, MA, USA). The expression levels of human endogenous retroviruses (HERVs), including HERV-K *env*, HERV-K *gag*, HERV-W *env*, and HERV-H *env*, as well as inflammatory cytokines (*IL-1*, *IL-6*, *TNF-*α), were measured using SYBR Green Master Mix (Applied Biosystems, Thermo Fisher Scientific, Waltham, MA, USA) and specific primers [21,22,23], synthesized by Thermo Fisher Scientific (Waltham, MA, USA) (Table 1). Since previous studies have shown that the *gapdh* gene is expressed consistently in various gravity settings [24,25], we used it as a housekeeping gene, and the 2^−ΔΔCT^ method was used to quantify gene expression.

### 2.4. Statistical Analysis

Statistical analysis was conducted using GraphPad Prism 8.0 software (GraphPad, San Diego, CA, USA). The clinostat experiment was performed three times, and the results present the average of these three independent experiments. Additionally, qPCR was performed in triplicates for each sample. Data distribution was analyzed using the D’Agostino–Pearson omnibus normality test and the Shapiro–Wilk test. Gene expression differences between microgravity (µg) and normal gravity (1g) conditions were analyzed using an unpaired two-sided Student’s t-test. If the data were normally distributed, Pearson’s correlation test was employed to evaluate the correlation between gene expression levels. Non-parametric data were analyzed using Spearman’s rank correlation test. A *p*-value of less than 0.05 was considered statistically significant.

## 3. Results

### 3.1. The Impact of Simulated Microgravity on Pro-Inflammatory Cytokine and HERV Gene Expression in SH-SY5Y, Caco-2, and HEp-2 Cells

**Figure 1:**
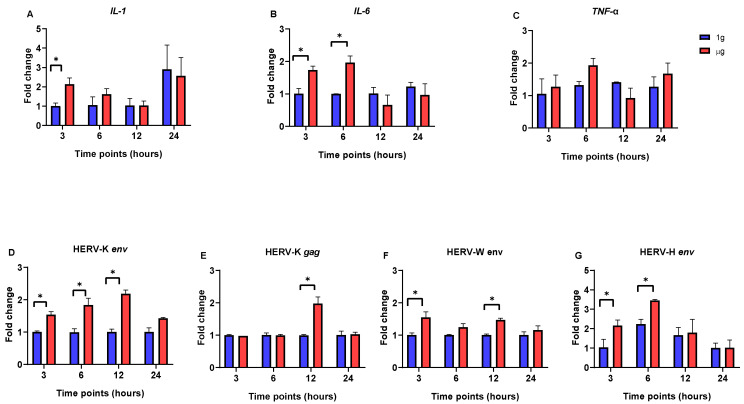
Quantitative real-time PCR analysis of HERV- and interleukin-related genes in SH-SY5Y cells at different time points (3, 6, 12, and 24 h) after incubation in µg and 1g conditions. (**A**) *IL-1*. (**B**) *IL-6*. (**C**) *TNF*-α. (**D**) HERV-K *env*. (**E**) HERV-K *gag*. (**F**) HERV-W *env*. (**G**) HERV-H *env*. Data are the average ± SD of two independent experiments. Stars above the bars indicate significant changes; *p* < 0.05.

We concentrated on the expression level of pro-inflammatory cytokines (*IL-1*, *IL-6*, and *TNF-a*) and HERV genes involved in neuro-inflammatory and cancer processes. In SH-SY5Y cells, the significant upregulation of *IL-1* and *IL-6* gene expression was observed after 3 h incubation in RPM-simulated µg conditions (Figure 1A,B). The expression of the *IL-6* gene remained highly elevated until the 6 h in µg, and then it began to decrease. By 12 and 24 h, the expression levels of both *IL-1* and *IL-6* in cells under μg conditions had returned to levels comparable to those observed under 1g conditions. The *TNF*-α expression level remained unchanged for all three time points (Figure 1C).

Moreover, the mRNA expression levels of HERV-K *env*, HERV-K *gag*, and HERV-W *env* were significantly upregulated in SH-SY5Y cells incubated under µg conditions for 12 h compared to the 1g-incubated cells (Figure 1D–F). Although the expression levels began to decrease after the 12 h in μg, they remained higher than those observed under 1g conditions. Furthermore, the expression of the HERV-H *env* gene was significantly increased in short incubation periods under µg conditions (3 and 6 h) (Figure 1G). However, after this initial increase, its expression decreased and remained at levels comparable to those observed under 1g conditions.

**Figure 2:**
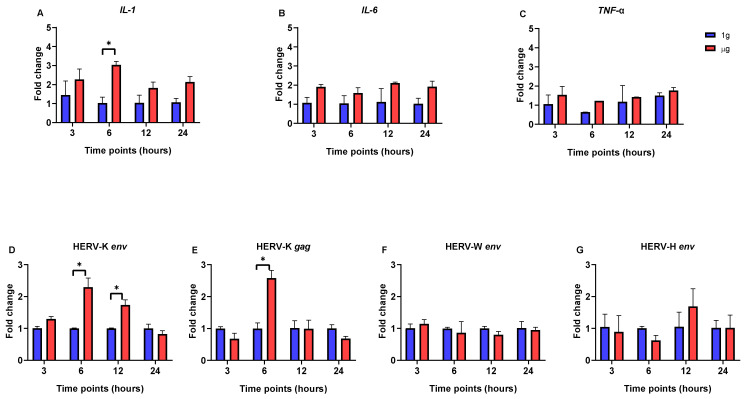
Quantitative real-time PCR analysis of HERV- and cytokine-related genes in Caco-2 cells at different time points (3, 6, 12, and 24 h) after incubation in µg and 1g conditions. (**A**) *IL-1*. (**B**) *IL-6*. (**C**) *TNF*-α. (**D**) HERV-K *env*. (**E**) HERV-K *gag*. (**F**) HERV-W *env*. (**G**) HERV-H *env*. Data are the average ± SD of two independent experiments. Stars above the bars indicate significant changes; *p* < 0.05.

In Caco-2 cells, while cytokine gene expression exhibited some fluctuations under µg conditions, no statistically significant differences were observed when compared to 1g conditions. The only exception was significant upregulation in the expression of the *IL-1* gene at the 6 h time point in the µg condition (Figure 2A,B).

Throughout the experiment, there were no significant changes in the expression levels of the HERV-W *env* and HERV-H *env* genes between Caco-2 cells incubated under µg conditions and those incubated under 1g conditions (Figure 2F,G). On the other hand, HERV-K gene expression was significantly upregulated in µg-exposed cells compared to the 1g control, and this increase continued for up to 12 h for HERV-K *env* and 6 h for the HERV-K *gag* gene (Figure 2D,E). After this, though, HERV-K’s expression level dropped and returned to what was observed in normal gravity.

**Figure 3:**
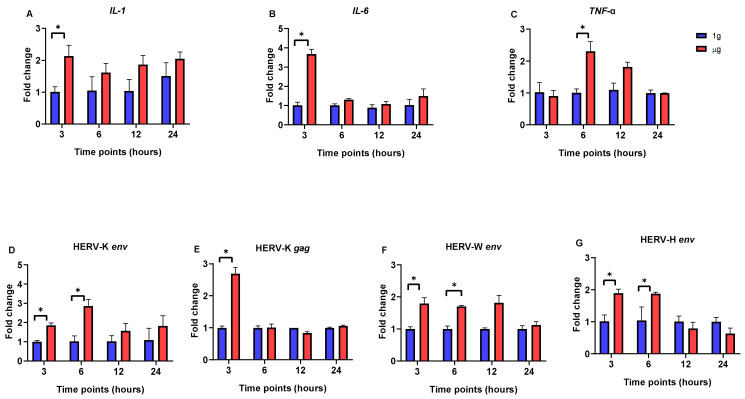
Quantitative real-time PCR analysis of HERV- and interleukin-related genes in HEp-2 cells at different time points (3, 6, 12, and 24 h) after incubation in µg and 1g conditions. (**A**) *IL-1*. (**B**) *IL-6*. (**C**) *TNF*-α. (**D**) HERV-K *env*. (**E**) HERV-K *gag*. (**F**) HERV-W *env*. (**G**) HERV-H *env*. Data are the average ± SD of two independent experiments. Stars above the bars indicate significant changes; *p* < 0.05.

In HEp-2 cells, the expression of the *IL-1* and *IL-6* genes was significantly upregulated following short-term exposure to µg (3 h) (Figure 3A,B). After this initial increase, the expression levels decreased and remained stable, similarly to those observed under 1g conditions. In parallel, the expression of the *TNF*-α gene was significantly upregulated at 6 h under µg conditions, with levels significantly higher compared to those observed under 1g conditions (Figure 3C).

In addition, the expression levels of four HERV genes were significantly increased after the 3 h (HERV-K *gag*) and 6 h (HERV-W *env*, HERV-K *env*, and HERV-H *env*) incubations under µg conditions, compared to those in 1g conditions (Figure 3D–G). After this point, though, these genes’ expression levels dropped, and there were no statistically significant changes between the µg and 1 g condition during the 12 and 24 h incubations.

These results demonstrate that microgravity changes the expression pattern of pro-inflammatory cytokines and HERVs in various cell types, particularly during 12 h of exposure, with each cell line exhibiting a distinct pattern of response.

### 3.2. Correlation Analysis of Gene Expression in Different Cell Lines

**Figure 4:**
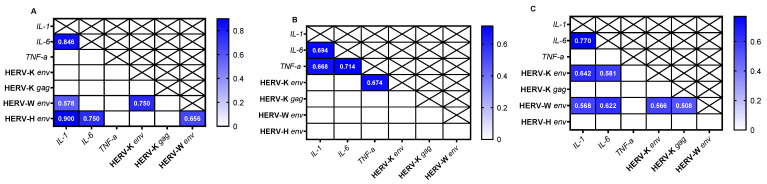
Heatmap shows the r values obtained from the Spearman correlation analysis of gene expression across different cell lines. (**A**) SH-SY5Y, (**B**) Caco-2, and (**C**) HEp-2 cell lines.

Finally, we evaluated the presence or absence of a correlation between the gene expression of HERVs and cytokines among different cell lines. Our analysis showed the presence of a positive correlation in SH-SY5Y cells between *IL-1* and HERV-H *env* (r = 0.900, *p* < 0.0001), *IL-1* and HERV-W *env* (r = 0.578, *p* < 0.02), and *IL-6* and HERV-H *env* (r = 0.750, *p* < 0.0012) (Figure 4A). In Caco-2 cells, a positive correlation was observed between *TNF*-α and HERV-K *env* (r = 0.674, *p* < 0.0052) (Figure 4B). For HEp-2 cells, there was a correlation between *IL-1* and HERV-W *env* (r = 0.568, *p* < 0.023), *IL-1* and HERV-K *env* (r = 0.642, *p* < 0.0081), *IL-6* and HERV-W *env* (r = 0.622, *p* < 0.011), and *IL-6* and HERV-K *env* (r = 0.581, *p* < 0.019) (Figure 4C).

## 4. Discussion

We examined the expression levels of most known pro-inflammatory cytokines (*IL-1*, *IL-6*, and *TNF-α*) and HERV genes linked to inflammatory disorders [26,27] in three different cell lines (human neuroblastoma SH-SY5Y cells, human laryngeal carcinoma HEp-2 cells, and human colorectal adenocarcinoma Caco-2 cells) under simulated μg and 1g conditions. In this study, in microgravity, the gene expression of pro-inflammatory cytokines *IL-1* and *IL-6* was elevated, particularly at the early time points (3 and 6 h), in the SH-SY5Y, Caco-2, and HEp-2 cell lines, indicating that microgravity induces an increase in pro-inflammatory factors across these cell lines. Previous studies have reported conflicting results regarding cytokine expression under simulated microgravity. Some studies demonstrated the upregulation of pro-inflammatory cytokines *IL-1*, *IL-6*, and *TNF-α* [28,29], whereas others showed a reduction in their expression levels in µg conditions [30,31]. For instance, in the study conducted by Dietrichs et al., after a 4 h exposure of prostate cancer cells (PC-3) to simulated microgravity, the expression level of *IL-1β*, *IL-6,* and *TNF-α* was elevated [32]. Conversely, it was also previously reported that the basal activation of the NF-κB pathway in Caco-2 cells (an intestinal in vitro model) was significantly reduced under μg conditions, resulting in impaired immune responses [33]. In another in vivo study, microgravity was shown to disrupt intestinal homeostasis, leading to an increase in the pro-inflammatory factor *IL-1* and a decrease in anti-inflammatory *IL-10* gene expression, which triggered susceptibility to colitis [34]. This variation in results is probably attributable to differences in experimental platforms, methodologies, and cell types, all of which require further investigation. In parallel, *TNF-*α expression displayed significant upregulation exclusively in HEp-2 cells after 6 h of exposure in µg. The differences in gene expression between cell lines suggest that individual cell types exhibit unique responses to microgravity, which requires further investigation.

Our results showed that microgravity affected HERV gene expression in three different cell lines. In SH-SY5Y and HEp-2 cells, the expression levels of HERV-W *env*, HERV-K, and HERV-H *env* were significantly upregulated after 3 h of incubation in the µg condition. However, in Caco-2 cells, only HERV-K *env* showed upregulation under µg compared to 1g conditions. The transient activation of HERVs in µg suggests that µg influences their expression, potentially impacting cellular immune responses. Interestingly, we detected the transcriptional activity of HERVs in all cell lines under 1g conditions. This observation could be explained by the fact that the cell lines used in our experiments were of neoplastic origin, which are known to exhibit a distinct transcriptional signature compared to normal cells [6,35].

Although no direct studies have been performed on HERV gene expression in microgravity conditions, previous studies have shown that elevated nitric oxide (NO) production in SH-SY5Y cells contributes to elevated oxidative stress [36,37]. The study conducted by Bi L et al. in 2009 examined the effects of parabolic flight conditions on the redox status of SHSY5Y cells. Their results demonstrated that under microgravity conditions, the levels of 3-nitrotyrosine (3-NT) and thioredoxin (TRX) increased, while the expression of thioredoxin reductase (TRXR) was reduced, suggesting an elevation in oxidative stress [37]. It is well established that oxidative stress is closely linked to inflammation and may significantly affect the activation of HERVs [35]. In our study, we observed an increase in inflammatory responses, along with higher expression of the HERV-W *env*, HERV-K *env*, and HERV-H *env* genes in SH-SY5Y cells under µg conditions, both of which have been reported as characteristic features of neuro-inflammation [12,38]. Interestingly, the Spearman analysis also demonstrated various positive correlations between HERV env mRNA levels and cytokines (*IL-1* and *IL-6*) across the cell lines, suggesting a significant interplay between inflammatory pathways and HERV activation.

However, our study has some limitations. The most obvious limitation of the current study was the focus on three cell lines which may not represent the diversity of HERV responses across different cell types. In addition, short-term exposure to simulated microgravity (up to 24 h) was another limitation. Furthermore, protein expression analyses of cytokines were not performed in this study, which could have provided further confirmation of the qPCR results. Therefore, future research needs to address these gaps by investigating the longer-term effects of µg, additional cell types, and the functional impacts of µg in gene expression studies of different HERV families with protein-level analyses.

## 5. Conclusions

In conclusion, we observed the upregulation of HERVs with varying patterns across different cell lines under microgravity conditions. This upregulation was correlated with the expression of pro-inflammatory cytokine genes. These preliminary findings provide valuable insights into space biology and human health during space missions.

## Figures and Tables

**Table 1:** The primers used in this study.

Target Gene	Sequence 5′ to 3′ (Forward)	Sequence 5′ to 3′ (Reverse)
*IL-1B*	F: GCACGATGCACCTGTACGAT	R: AGACATCACCAAGCTTTTTTGCT
*IL-6*	F: CCAGGAGCCCAGCTATGAAC	R: GAGCAGCCCCAGGGAGAA
*TNF-α*	F: CAGAGGGAAGAGTTCCCCAG	R: CCTTGGTCTGGTAGGAGACG
GAPDH	F: CAAGGAGTAAGACCCCTGGAC	R: TCTACATGGCAACTGTGAGGAG
HERV-H *env*	F: CCCATATTTGGACCTCTCAC	R: TGTGTAGTTGGGCTTTGGAG
HERV-W *env*	F: CCTATTTAATACCACCCTCACTG	R: AGTTGTTCCATTGTTCAGGT
HERV-K *env*	F: GCTGTCTCTTCGGAGCTGTT	R: CTGAGGCAATTGCAGGAGTT
HERV-K *gag*	F: CCCATGGTTTCCAGAACAAGG	R: AAGCTGCTTTAATAATGGCCC

## Data Availability

The original contributions presented in this study are included in the article. Further inquiries can be directed to the corresponding author.
